# Comparative Genomic Analysis of Re-emergent Human Adenovirus Type 55 Pathogens Associated With Adult Severe Community-Acquired Pneumonia Reveals Conserved Genomes and Capsid Proteins

**DOI:** 10.3389/fmicb.2018.01180

**Published:** 2018-06-05

**Authors:** Zetao Cheng, Yuqian Yan, Shuping Jing, Wen-Gang Li, Wei-Wei Chen, Jing Zhang, Min Li, Shan Zhao, Na Cao, Junxian Ou, Suhui Zhao, Xianbo Wu, Bin Cao, Qiwei Zhang

**Affiliations:** ^1^Guangzhou Key Laboratory of Drug Research for Emerging Virus Prevention and Treatment, Guangdong Provincial Key Laboratory of Tropical Disease Research, School of Public Health, Southern Medical University, Guangzhou, China; ^2^Treatment and Research Center for Infectious Diseases, 302 Military Hospital of China, Beijing, China; ^3^Department of Pulmonary and Critical Care Medicine, China-Japan Friendship Hospital, Beijing, China; ^4^Dermatology Hospital, Southern Medical University, Guangzhou, China

**Keywords:** human adenovirus type 55, recombination, comparative genomics, severe community-acquired pneumonia, China

## Abstract

Human adenovirus type 55 (HAdV-B55) is a recently identified acute respiratory disease (ARD) pathogen in HAdV species B with a recombinant genome between renal HAdV-B11 and respiratory HAdV-B14. Since HAdV-B55 first appeared in China school in 2006, no more ARD cases associated with it had been reported until 2011, when there was an outbreak of adult severe community-acquired pneumonia (CAP) in Beijing, China. Reported here is the bioinformatics analysis of the re-emergent HAdV-B55 responsible for this outbreak. Recombination and protein sequence analysis re-confirmed that this isolate (BJ01) was a recombinant virus with the capsid hexon gene from HAdV-B11. The selection pressures for the three capsid proteins, *i.e.,* hexon, penton base, and fiber genes, were all negative, along with very low non-synonymous (dN) and synonymous (dS) substitutions/site (<0.0007). Phylogenetic analyses of the whole genome and the three major capsid genes of HAdV-B55 revealed the close phylogenetic relationship among all HAdV-B55 strains. Comparative genomic analysis of this re-emergent HAdV-B55 strain (BJ01; 2011) with the first HAdV-B55 strain (QS-DLL; 2006) showed the high genome identity (99.87%), including 10 single-nucleotide non-synonymous substitutions, 11 synonymous substitutions, 3 insertions, and one deletion in non-coding regions. The major non-synonymous substitutions (6 of 10) occurred in the protein pVI in its L3 region, which protein has different functions at various stages of an adenovirus infection, and may be associated with the population distribution of HAdV-B55 infection. No non-synonymous substitutions were found in the three major capsid proteins, which proteins are responsible for type-specific neutralizing antibodies. Comparative genomic analysis of the re-emergent HAdV-B55 strains associated with adult severe CAP revealed conserved genome and capsid proteins, providing the foundation for the development of effective vaccines against this pathogen. This study also facilitates the further investigation of HAdV-B55 epidemiology, molecular evolution, patterns of pathogen emergence and re-emergence, and the predication of genome recombination between adenoviruses.

## Introduction

Human adenoviruses (HAdV) are associated in humans with a broad spectrum of clinical diseases, such as acute respiratory disease (ARD), community-acquired pneumonia (CAP), as well as conjunctivitis, gastrointestinal, opportunistic infections in immune-deficient individuals, and possibly, obesity (Dingle and Langmuir, 1968; Chu and Pavan-Langston, 1979; Wood, 1988; Dhurandhar et al., 1992; Kojaoghlanian et al., 2003; Hakim and Tleyjeh, 2008). Since the first HAdV isolate was found in 1953 (Hilleman et al., 1953; Rowe et al., 1953), more than 80 HAdV genotypes have been identified and classified into seven species (A to G) using the whole-genome sequence as the typing standard^1^ (Jones et al., 2007; Ishiko et al., 2008; Walsh et al., 2009, 2010, 2011; Liu et al., 2011, 2012; Matsushima et al., 2011, 2012, 2013; Robinson et al., 2011, 2013b; Seto et al., 2011; Dehghan et al., 2012; Singh et al., 2012, 2013; Zhou et al., 2012; Alissa Alkhalaf et al., 2014; Hage et al., 2015; Hashimoto et al., 2018). HAdV species B are further divided into subspecies B1 (HAdV-B3, -B7, -B16, -B21, and -B50) and B2 (HAdV-B11, -B14, -B34, -B35, and -B55) according to their restriction enzyme digestion patterns (Wadell et al., 1980). Subspecies B1 cause respiratory disease except for HAdV-B50. The members in subspecies B2 cause renal or urinary tract disease, except for HAdV-B14 and -B55, which are associated with ARD (Echavarria, 2008).

Human adenoviruses are usually typed according to serum neutralization and hemagglutination-inhibition tests, both of which are associated with the three major capsid proteins, *i.e*., hexon, fiber, and penton base. Based on partial characterization of its hexon and fiber epitopes, as well as the restriction enzyme patterns (Zhang Q. et al., 2016), HAdV-B55 was first identified as HAdV-B11a (Hierholzer et al., 1974). This virus may have occurred earlier from an ARD outbreak in a military training base (Spain, 1969), and subsequently re-emerged in several outbreaks of ARD in Turkey (2004), Singapore (2005) discontinuously (Hierholzer et al., 1974; Chmielewicz et al., 2005; Zhu et al., 2009; Kajon et al., 2010; Li et al., 2014; Lu et al., 2014). Complete genome analysis suggested that this new type, HAdV-B55, is a Trojan horse, which contains a recombinant genome from both a renal pathogen, HAdV-B11, which provides the antigenic epitope, and a respiratory pathogen, HAdV-B14, which confers the cell tropism, biological and pathogenicity properties (Walsh et al., 2010). The strain QS-DLL is the first completely characterized HAdV-B55 based on the whole genomic analysis (Walsh et al., 2010). It caused an ARD outbreak in China, from March to April 2006, with 254 patients affected, of which 247 were students in a senior high school and one died during this outbreak (Zhu et al., 2009).

Since this first isolate QS-DLL of HAdV-B55 was identified in 2006 (Yang et al., 2009; Walsh et al., 2010), no more ARD cases associated with this virus had been reported in China until 2011, when 16 HAdV-B55-like strains were identified based on partial gene sequencing in a CAP outbreak in Beijing, China in February and March (Cao et al., 2014). Of these, strain BJ01 was isolated from a throat swab of a 29-year-old adult who presented with severe CAP (Gu et al., 2012). The whole genome (34,773 bp) of strain BJ01 (Human adenovirus B human/CHN/BJ01/2011/55[P14H11F14]) was sequenced using the Sanger method, following PCR amplification of targeted overlapping regions. (GenBank accession number JX491639). (Zhang et al., 2012a). In this study, comparative genomic analysis of this re-emergent HAdV-B55 strain (BJ01) with the first HAdV-B55 strain (QS-DLL), as well as the bioinformatic analysis of HAdV-B55 strains circulating recently were performed. The findings will contribute to the studies of the evolution and epidemiology of HAdV-B55, as well as provide both the foundation for the development of effective vaccines and public health strategies against this pathogen.

## Materials and Methods

### Cells, Virus Stock, and Genomic DNA Extraction

This specimen was originally isolated from a throat swab of an adult diagnosed with severe CAP at Beijing Chao-Yang Hospital of China (March 2011) (Gu et al., 2012; Zhang et al., 2012a). Written informed consent was obtained from the patient. The protocol was approved by the Ethics Committee of Beijing Chao-Yang Hospital, Capital Medical University in accordance with the Declaration of Helsinki. The throat swab was inoculated and cultured in A549 cells (ATCC), and grown in Dulbecco’s minimum essential medium supplemented with 100 IU/ml penicillin, 100 μg/ml streptomycin, and 2% (v/v) fetal calf serum, at an atmosphere of 5% (v/v) carbon dioxide. Viral genomic DNA was extracted from infected A549 cells, as described earlier (Le et al., 2001). PCR amplification and molecular typing was performed according to the previous study (Han et al., 2013).

### Genome Sequencing and Annotation

The genome of strain BJ01 was sequenced using a Sanger primer-walking method, following PCR amplification of targeted overlapping regions (Zhang et al., 2012a,b). Both 5^′^- and 3^′^-ends (including both inverted terminal repeats) were sequenced directly by 5^′^ primer Ad14-LTRS1 (5^′^-ACAGAAGACTTTCACACGGT-3^′^) and 3^′^ primer Ad14-LTRS2 (5^′^-GGTCCCTCTAAATACACATACA-3^′^), respectively, using extracted genomic DNA as sequencing template. The sequence data provided average three- to fivefold coverage of the genome. Ambiguous sequences and gaps were re-amplified and re-sequenced. All the sequencing ladders were assembled using the SEQMAN (DNAStar; Madison, WI). The genome was further annotated according to the previous report (Walsh et al., 2010).

### Genome Recombination Analysis

Genome sequence recombination analysis was done using Simplot 3.5.1^2^, including the options of Simplot and Bootscan analysis (Lole et al., 1999). The software MAFFT was used to align the HAdV-B sequences^3^. The parameters of gap opening penalty (1.53), Offset value (0), and the Scoring matrix for nucleotide sequences (200 PAM/k = 2) were set by default. The LAGAN (Limited Area Global Alignment of Nucleotides) program of mVISTA was used for the pair-wise comparisons of genomes^4^ (Brudno et al., 2003).

### Phylogenetic Analysis

Phylogenetic analysis was performed by Molecular Evolutionary Genetics Analysis (MEGA) 5.0.5^5^ (Tamura et al., 2007). Phylogenetic trees with 1,000 bootstrap replications were constructed using a maximum-composite-likelihood method. All the other parameters were set by default.

### Substitution Rate Analysis of HAdV-B55 Hexon, Penton Base, and Fiber Genes

The numbers of non-synonymous (dN) and synonymous (dS) substitutions per site between sequences and dN/dS ratios were calculated. The analysis was performed with MEGA 5.05 (Tamura et al., 2011) using the Nei–Gojobori model (Nei and Gojobori, 1986). The nucleotide sequences from 23 hexon, 43 fiber, and 17 penton base genes available in GenBank were included.

All the genome sequences included for substitution rate analysis were summarized in **Table 1**. For HAdV-B55, additional genome typing details were also presented. The sequences of the hexon, penton base, and fiber genes were either extracted from the genome files or deposited as single gene entries.

**Table 1:** The genome, hexon, penton base and fiber sequences of adenovirus species A-G used in this study.

Type	Strain	Year isolated	Country	Sequence	GenBank accession no.
HAdV-A12	Huie	1954	USA	Genome	AC_000005
HAdV-B3	GB	1953	USA	Genome	AY599834
HAdV-B7	Gomen	1952	USA	Genome	AY594255
HAdV-B11	Slobitski	1956	USA	Genome	NC_011202
HAdV-B11a^∗^	ak37	2001	EGY	Genome	JX423385
	1222	2005	SGN	Genome	FJ597732
	AK36	2005	ARG	Genome	JX423384
HAdV-B14	de Wit	1955	Netherlands	Genome	AY803294
HAdV-B55	BJ01^#^	2011	CHN	Genome	JX491639
	CQ-814	2010	CHN	Genome	JX123027
	BD6728	2013	CHN	Genome	KJ883520
	TJ-2013-90	2013	CHN	Genome	KF908851
	CQ-2903	2012	CHN	Genome	JX123029
	QS-DLL	2006	CHN	Genome	FJ643676
	BD6729	2013	CHN	Genome	KJ883521
	QZ01	2011	CHN	Genome	KJ883522
	CQ-1657	2011	CHN	Genome	JX123028
	XZ2012-492	2012	CHN	Genome	KC857701
	Hebei/BD01	2012	CHN	Genome	KP896478
	Liaoning/LS01	2013	CHN	Genome	KP896483
	Tianjin/TJ01	2013	CHN	Genome	KP896484
	JS201501	2015	CHN	Genome	KX289874
	60-GD-2016	2016	CHN	Genome	KY070248
	AFMC 16-0011	2016	S. Korea	Genome	KX494979
	AH-CHN/CZ-TC8	2012	CHN	Hexon	KC551973
	HAdV11-QS	2010	CHN	Hexon	DQ874353
	SHX-P01	2011	CHN	Hexon	KC999882
	Shanxi-Y16	2011	CHN	Hexon	KF911353
	BJ10	2013	CHN	Hexon	KM458628
	87	2016	CHN	Fiber	KY070249
	60	2016	CHN	Fiber	KY070250
	59	2016	CHN	Fiber	KY070251
	81	2016	CHN	Fiber	KY070252
	111	2016	CHN	Fiber	KY070253
	161	2016	CHN	Fiber	KY070254
	169	2016	CHN	Fiber	KY070255
	73	2016	CHN	Fiber	KY070256
	123	2016	CHN	Fiber	KY070257
	80	2016	CHN	Fiber	KY070258
	BJ10	2013	CHN	Fiber	KP270921
	YT2011_12-44	2011	CHN	Fiber	KC510748
	YT2011_12-115	2011	CHN	Fiber	KC510749
	BJ2011_6-80	2011	CHN	Fiber	KC510750
	YT2011_12-31	2011	CHN	Fiber	KC510751
	BJ2011_10-54	2011	CHN	Fiber	KC510752
	BJ2011_7-35	2011	CHN	Fiber	KC510753
	BJ2011_10-25	2011	CHN	Fiber	KC510754
	BJ2011_1-32	2011	CHN	Fiber	KC510755
	BJ2011_4-28	2011	CHN	Fiber	KC510756
	BJ2011_10-39	2011	CHN	Fiber	KC510757
	BJ2011_1-31	2011	CHN	Fiber	KC510758
	BJ2011_1-29	2011	CHN	Fiber	KC510759
	BJ2011_1-100	2011	CHN	Fiber	KC510760
	BJ2011_1-98	2011	CHN	Fiber	KC510761
	YT2011_12-42	2011	CHN	Fiber	KC510762
HAdV-B16	ch.79	1955	USA	Genome	AY601636
HAdV-B21	AV-1645	1956	Saudi Arabia	Genome	AY601633
SAdV-21	Bertha	1954	USA	Genome	AC_000010
HAdV-B34	Compton	1972	USA	Genome	AY737797
HAdV-B35	Holden	1973	USA	Genome	AY128640
HAdV-B50	Wan	1988	USA	Genome	AY737798
HAdV-B66	87-922	1987	ARG	Genome	JN860676
HAdV-B68	Arg 827/04	2004	ARG	Genome	JN860678
HAdV-C1	Adenoid 71	1953	USA	Genome	AF534906
HAdV-D9	Hicks	1954	USA	Genome	AJ854486
HAdV-E4	RI-67	1952	USA	Genome	AY594253
HAdV-F40	Dugan	1979	Netherlands	Genome	NC_001454
HAdV-G52	T03-2244	2003	USA	Genome	DQ923122

*They are either deposited as single gene entries in GenBank or extracted from the genome files. ^#^The HAdV-B55 genome analyzed in the present study. ^∗^They are likely typed as HAdV-B55 according to the genome sequences.*

### Sequences Used in the Study

The HAdV sequences of the hexon, penton base, fiber genes, and the genomes used for phylogenic analyses were retrieved from GenBank and summarized in **Table 1**, with additional sequence origin details if available (strain names, countries, years and GenBank accession numbers). The prototype strains were also used.

## Results

### Nucleotide Sequence Analysis of HAdV-B55 Strain BJ01

The genome data of strain BJ01 was deposited into GenBank (accession number JX491639), and named formally as “Human adenovirus 55 isolate HAdV-B/CHN/BJ01/2011/55[P14H11F14]”, further referred to as “BJ01”.

**FIGURE 1:**
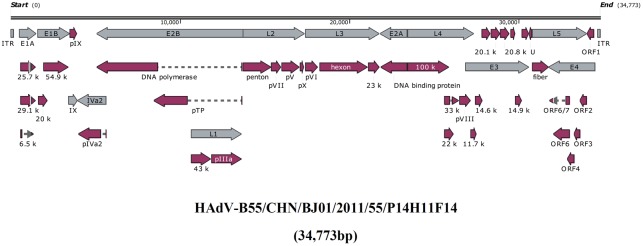
Transcriptional map and genome organization of HAdV-B55 strain BJ01. The genome is indicated by two black horizontal lines marked at 10,000 bp intervals. Early, intermediate, and late transcription units are designated by gray arrows, while the red designates coding regions. Arrows reflect the transcriptional orientation of the coding transcripts.

**Figure 1** presented the genomic organization and transcription map of strain BJ01. The genome contained four early, two intermediate, and five late transcription units, and 34,773 bp in length, with a base composition of 26.16% A, 25.08% T, 24.38% G, 24.39% C, and a GC content of 48.76%, which was similar with the other members of subspecies B2 (mean of 49%) (Seto et al., 2010a). A total of 39 coding sequences were identified (**Table 2**). The 137-bp ITR of strain BJ01 was identical to that of the first HAdV-B55 strain (QS-DLL; 2006) (Walsh et al., 2010).

**Table 2:** The genome sequence annotation of HAdV-B55 strain BJ01.

Region	Product	Location
E1A	29.1 kDa protein	58–1166,1251–1459
E1A	25.7 kDa protein	587–1073,1251–1459
E1A	6.5 kDa protein	587–658,1251–1355
E1B	20 kDa protein	1629–2171
E1B	54.9 kDa protein	1934–3418
pIX	pIX protein	3501–3920
IVa2	IVa2 protein	3989–5322,5601–5613
E2B	DNA polymerase	5092–8664,13645–13653
E2B	pTP	8463–10424,13645–13653
L1	43 kDa protein	10671–11831
L1	pIIIa	11857–13620
L2	penton base protein	13701–15374
L2	protein VII	15379–15957
L2	protein V precursor	16000–17055
L2	protein X	17084–17314
L3	protein VI	17395–18135
L3	hexon protein	18251–21091
L3	23 kDa protein	21131–21760
E2A	DNA binding protein	21838–23394
L4	100 kDa hexon-assembly associated protein	23425–25863
L4	33 kDa protein	25595–25913,26083–26444
L4	22 kDa protein	25595–26170
L4	protein VIII	26494–27177
E3	11.7 kDa protein	27177–27494
E3	14.6 kDa protein	27448–27843
E3	18.4 kDa protein	27828–28328
E3	20.1 kDa protein	28348–28893
E3	20.8 kDa protein	28911–29462
E3	10.1 kDa protein	29506–29781
E3	14.9 kDa protein	29786–30190
E3	15 kDa protein	30183–30590
U	U protein	30614..30778
L5	fiber protein	30793–31770
E4	Orf6/7 protein	31806–32057,32780–32953
E4	Orf6 protein	32054–32953
E4	Orf4 protein	32856–33224
E4	Orf3 protein	33233–33586
E4	Orf2 protein	33583–33972
E4	Orf1 protein	34017–34394

### Protein Sequence Analysis of HAdV-B55 Strain BJ01

The amino acid sequence percent identities of the proteins of HAdV-B55 strain BJ01 were presented in **Table 3**. Selected proteins of the representative prototypes in each HAdV species, including all species B adenoviruses, were used for comparison. The amino acid sequence analysis revealed that the proteins of strain BJ01 had the highest amino acid percent identities with that of HAdV-B14 (strain de Wit) except for the hexon protein, which is responsible for virus characterization by serum neutralization. The hexon protein of strain BJ01 had the highest amino acid identity with HAdV-B11 (98.4%), rather than HAdV-B14 (92.2%). The amino acid sequence identities indicated that strain BJ01 is a recombinant between HAdV-B14 and HAdV-B11, with the HAdV-B11 hexon gene inserted chimeric into the HAdV-B14 genome backbone (**Table 3**). Interestingly, E3 11.7 kDa protein of HAdV-B55 strain BJ01 had the same amino acid sequence identity with both HAdV-B14 and HAdV-B35, indicating that these three types share the conserved and similar E3 11.7 kDa protein. Among all the proteins analyzed, the highest sequence diversity was found in the fiber gene of all the seven species.

**Table 3:** Amino acid percent identities of select HAdV-55 strain BJ01 proteins with representative HAdVs from all species, including all species B adenoviruses.

Protein	E1A	E1B	E2B	L1	L2	L3	E2A	L4	E3	L5	E4
	**29.1 kDa**	**20 kDa**	**DNA polymerase**	**pIIIa**	**Penton base**	**Hexon**	**DBP**	**pVIII**	**11.7 kDa**	**Fiber**	**ORF6**

HAdV-B35	95.8	98.3	93.2	99.5	98.6	94.4	62.9	99.1	**99.1**	62.1	97.7
HAdV-B34	97.7	99.4	98.7	99.3	95.3	91.3	99.4	99.1	98.1	62.2	97.7
HAdV-B14	**97.7**	**100.0**	**99.7**	**99.8**	**99.5**	92.2	**99.8**	**99.6**	**99.1**	**99.1**	**99.3**
HAdV-B11	96.6	98.3	93.0	99.3	98.4	**98.4**	99.2	99.1	98.1	92.3	98.0
HAdV-B50	79.4	88.9	90.8	91.2	91.8	90.7	83.4	94.7	89.6	61.5	96.0
HAdV-B21	79.4	89.0	90.6	90.3	92.0	92.4	83.4	94.3	89.6	61.5	96.0
HAdV-B16	79.8	88.9	91.0	92.9	85.2	85.3	83.4	94.7	89.6	51.4	89.0
HAdV-B7	79.4	87.8	89.9	92.7	85.3	86.6	82.6	94.3	90.6	91.1	96.7
HAdV-B3	79.0	87.2	89.9	93.0	85.5	86.3	83.8	94.3	89.6	56.7	97.3
SAdV-B21	79.8	87.9	91.5	92.0	91.5	90.5	89.0	95.2	89.6	55.2	84.1
HAdV-B66	79.4	87.8	90.7	92.9	85.5	86.3	82.4	94.7	90.6	56.1	97.0
HAdV-B68	69.5	88.9	90.7	92.9	85.2	85.7	83.2	94.7	89.6	51.4	89.0
HAdV-A12	42.4	42.2	70.2	71.0	72.2	76.8	47.1	77.4	61.0	16.8	49.5
HAdV-C1	36.3	49.2	74.0	74.5	68.4	76.6	55.3	79.7	51.4	17.5	9.3
HAdV-D9	41.6	53.0	74.3	79.0	76.3	81.2	59.8	80.6	65.1	26.3	64.6
HAdV-E4	54.7	60.0	84.7	86.7	82.7	82.3	72.7	89.9	74.5	23.0	70.0
HAdV-F40	38.1	46.1	70.1	70.4	72.5	78.6	45.0	79.0	14.8	20.6	45.3
HAdV-G52	37.7	46.6	72.4	70.5	72.7	78.7	48.5	77.7	57.1	24.6	48.0

*The proteins above span the whole genome. The highest values from each selected protein are in bold*.

### Nucleotide Substitution Rates and Selection Pressures for HAdV-B55 Major Capsid Protein Genes

The synonymous and non-synonymous substitutions as well as the selection pressures for the three major capsid protein genes of HAdV-B55 were examined and compared. All the dN/dS ratios of the three genes were less than 1 (**Table 4**). This is to be expected because the evolution is dominated by negative selection, which removes mutations harmful to fitness (Zhao et al., 2014). Specifically, the hexon gene had least non-synonymous substitutions per site and the lowest ratio of dN/dS (0.06254). The non-synonymous substitutions and dN/dS ratio of the fiber gene was also low. However, it was relatively higher than that of the penton base and hexon genes. Overall, the major capsid proteins of HAdV-B55, hexon, penton base, and fiber, are highly conserved when compared with other types, such as HAdV-B7 (Zhao et al., 2014).

**Table 4:** Nucleotide substitution rate and selection pressures for the HAdV-55 major capsid protein genes.

Gene	Length(bp)	No. of sequences	Mean Non-synonymous Substations/site	Mean Synonymous Substitutions/site	dN/dS
Hexon	2839	23	0.00004	0.00064	0.06254
Penton base	1674	17	0.00048	0.00059	0.81874
Fiber	978	43	0.00037	0.00039	0.96781

*The mean numbers of non-synonymous (dN) and synonymous(dS) substitutions per site from between sequences were noted and dN/dS were calculated. This selection pressure analysis was conducted using the maximum-composite-likelihood model, and included nucleotide sequences from 23 hexon, 43 fiber, and 17 penton base genes of HAdV-55 available from GenBank. All positions containing gaps were eliminated automatically. Evolutionary analyses were performed with MEGA 5.05 version*.

### Genome Recombination Analysis of HAdV-B55 Strain BJ01

**FIGURE 2:**
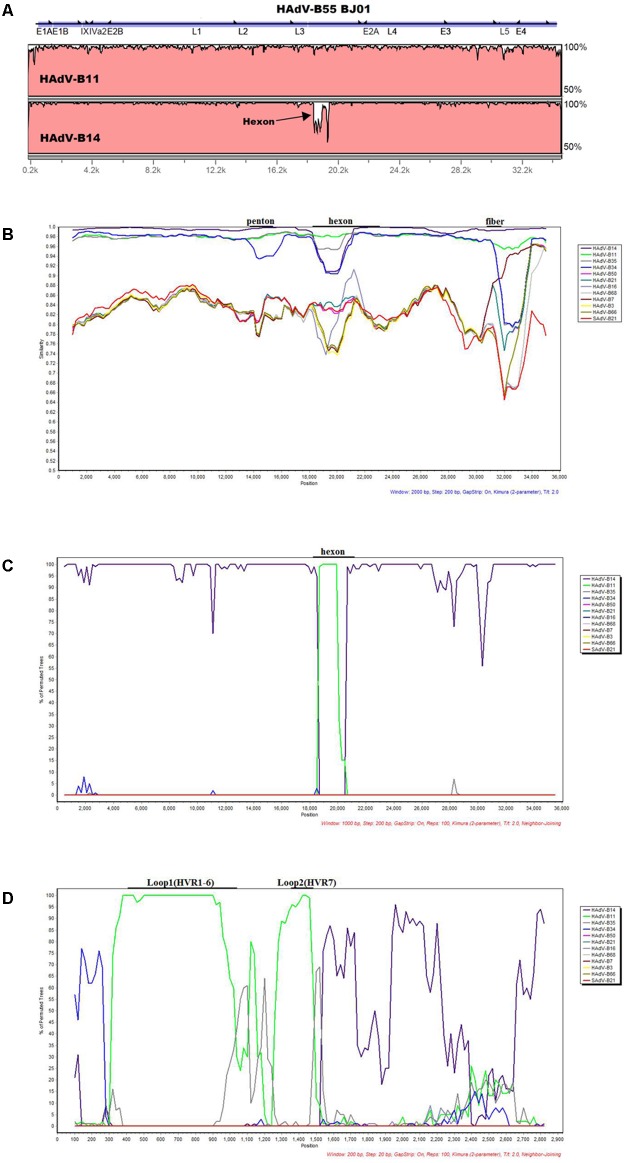
Genome and recombination analysis of HAdV-B55 Strain BJ01. **(A)** Global pairwise alignment of HAdV-B55 (BJ01) with HAdV-B11 and HAdV-B14 using mVISTA LAGAN. Transcription units are shown above the graph by black arrows relative to their position and orientation in the HAdV genome. The y-axis represents the sequence similarity and the genome positions are indicated on the x-axis. **(B)** Simplot and **(C)** Bootscan analysis of the complete genomes of strain BJ01 compared to other species B adenoviruses. **(D)** Bootscan analysis of the hexon genes compared with other HAdV-B types. Parameters are as follows: 100 repetitions; Kimura distance model; neighbor-joining tree model; **(B)** 2,000-bp window, 200-bp step; **(C)** 1,000-bp window, 200-bp step; **(D)** 200-bp window, 20-bp step. Genome nucleotide positions are noted along the x-axis, and the sequence similarities are indicated along the y-axis. The landmarks above each graph demonstrate the approximate positions of the major capsid protein genes. **(B)/(C)/(D)**: Main genomes used were marked in the graph. Colors: violet, HAdV-B14; bright green, HAdV-B11; dark gray, HAdV-B35; blue, HAdV-B34; pink, HAdV-B50; blue green, HAdV-B21; gray-violet, HAdV-B16; light gray, HAdV-B68; dull-red, HAdV-B7; yellow, HAdV-B3, brown, HAdV-B66; bright red, SAdV-B21.

The software mVISTA-LAGAN was used to compare the genome sequence identity between HAdV-B55 strain BJ01, HAdV-B11, and -B14. The visualization alignment showed that HAdV-B55 BJ01 had higher genome sequence identity with HAdV-B14 (98.879%) globally than with HAdV-B11 (97.605%), except for the L3 region (the hexon gene), where strain BJ01 had higher sequence identity with HAdV-B11 than HAdV-B14 (**Figure 2A**). This indicates that HAdV-B55 BJ01 was a recombinant composed of the HAdV-B14 genome backbone and the chimeric HAdV-B11 hexon.

Recombination analysis of both the genome and the hexon gene of HAdV-B55 strain BJ01 along with other HAdV-B viruses were shown in **Figures 2B–D**. Simplot analysis strongly indicates that there was a recombination event between HAdV-B14 and HAdV-B11 (**Figure 2B**). Apparently, except for the hexon region, BJ01 had the highest similarity with HAdV-B14 instead of HAdV-B11 (**Figure 2B**). Bootscan analysis of the genome further confirmed the recombination (**Figure 2C**). The fine-resolution Bootscan analysis of the divergent hexon genes showed that the recombination event was within the hexon gene. Loop 1 (HVR1–6; nt 407 to 1,034) and Loop 2 (HVR7; nt 1,363 to 1,484) were derived from HAdV-B11 (the green regions). The other portions were derived from HAdV-B14, so was the rest of the genome (**Figure 2D**).

### Phylogenetic Analysis of the Whole Genome, the Hexon, Penton Base, and Fiber Genes of the HAdV-B55

**FIGURE 3:**
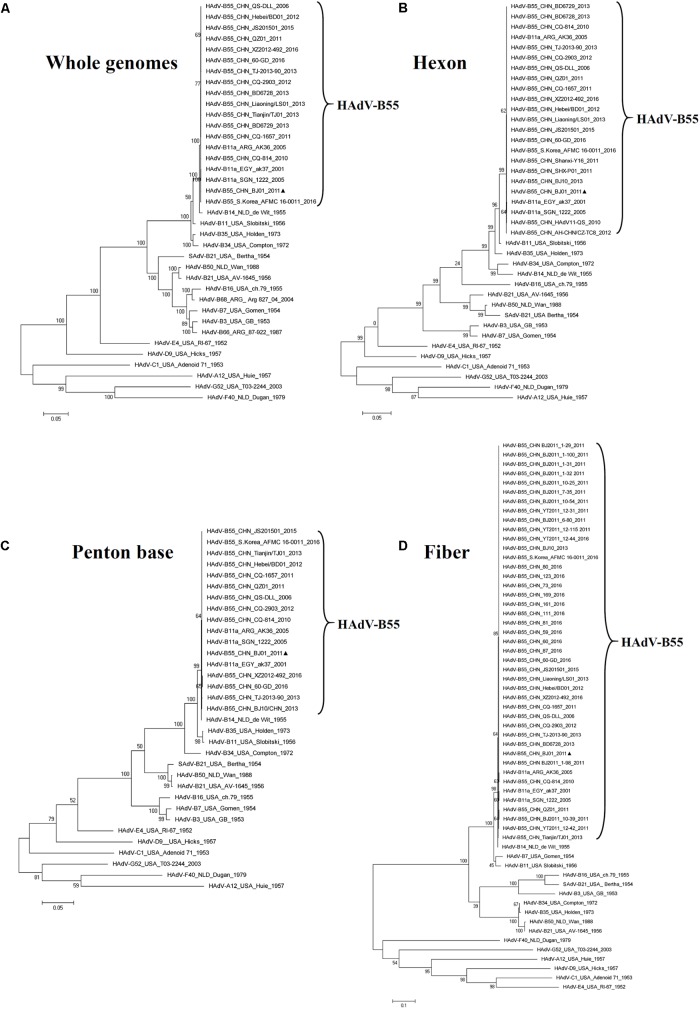
Phylogenetic analysis of HAdV-B55 strain BJ01. The HAdV nucleotide sequences of the whole genome **(A)**, hexon **(B)**, penton base **(C)**, and fiber **(D)** genes, were analyzed for their phylogenetic relationships. For reference, taxon names include the corresponding GenBank accession number, country of isolation, strain name, year of isolation, and genome type. Bootstrapped, maximum likelihood trees with 1,000 replicates were constructed using the MEGA 5.05 software (see Footnote 5). and by applying default parameters, with a maximum-composite-likelihood method. Bootstrap numbers shown at the nodes indicate the percentages of 1,000 replications producing the clade. The scale bar indicates units of nucleotide substitutions per site. The sequences used for phylogenic analyses are retrieved from GenBank and summarized in **Table 1**. Sequences from strain BJ01 are noted for reference (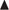).

The phylogenetic analysis of the whole genomic sequences confirmed that the genomes of HAdV-B members were closely related to each other, forming a clade (**Figure 3A**). HAdV-B55 was closer to HAdV-B14 than HAdV-B11 genome (**Figure 3A**), which was consistent with the global pairwise alignment result in **Figure 2A** and the genome percent identities between the two types (98.879% vs 97.605%). The phylogenetic analysis of the fiber base and penton base genes drew the same conclusion as the whole genomes: they were closer to HAdV-B14 than HAdV-B11 (**Figures 3C,D**). However, the phylogenetic tree of the hexon genes showed a different distribution: HAdV-B55 was closer to HAdV-B11 than HAdV-B14 (**Figure 3B**), which was consistent with the Bootscan analysis in **Figures 2C,D** as well as with the amino acid percent identities of the hexon protein (the highest identity with HAdV-B11: 98.4%) (**Table 3**).

### Comparative Genomic Analysis of the First Two HAdV-B55 Strains Emerged in China: QS-DLL and BJ01

Comparative genomics analysis showed that strain BJ01 had a high genome identity with strain QS-DLL (99.87%). However, when compared between the two genomes in high resolution detail, 22 single-nucleotide substitutions, 3 insertions and one deletion were identified between the two genomes. (**Table 5**).

**Table 5:** Comparative genomics analysis of two HAdV-B55 strains: QS-DLL and BJ01.

Region	Product	Position	Mutation in DNA	Substitution in protein	
				Non-synonymous	Synonymous
E1A	NCR	1463	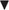 TCCATATCCGTGT	–	–
E1B	20kDa	1721	A→T	I→L	–
		1885	T→C	–	A→A
	55kDa	2170	T→C	S→P	–
		2823	A→G	–	K→K
IX	NCR	3910	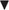 AAAAAA	–	–
E2B	DNA polymerase	8352	A→G	–	|→|
	pTP	10309	G→A	P→S	–
L1	43kDa	11293	G→A	–	A→A
L2	pV	16892	C→T	–	R→R
	NCR	17310-17311	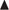 AA	–	–
	NCR	17328	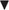 A	–	–
	NCR	17374	G→A	–	–
L3	pVI	17383	G→A	*D→N*	–
		17573	CG	*A→G*	–
		17579	GA	*R→K*	–
		17598	GA	–	Q→Q
		17617	GT	*V→F*	–
		17643	TG	*N→K*	–
		17676	GC	*Q→H*	–
	Hexon	19861	T→C	–	L→L
E2A	DNA Binding Protein	22846	A→C	–	L→L
L4	Hexon-assembly Associated Protein	25509	A→G	–	L→L
E3	11.7kDa	27198	T→C	–	H→H
	20.1kDa	28827	G→A	–	K→K
	14.9kDa	30017	G→T	A→S	–

*Nucleic acid and amino acid sequence changes are noted, along with their genome locations and coding consequences. The reference genome is HAdV-B55 strain QS-DLL. NCR, non-coding region; 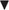, insertion; 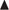, deletion; –, no change or not applicable. The major non-synonymous substitutions in pVI are italicized*.

**FIGURE 4:**
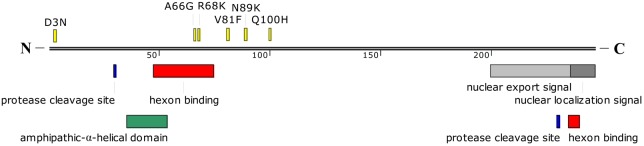
Schematic representation of HAdV-B55 protein VI. The amino acid mutations between HAdV-B55 strains BJ01 and QS-DLL are indicated in yellow. Sites for cleavage by the protease, nuclear export signal, and nuclear localization signal are also indicated in different colors. The hexon binding domains are located between amino acid residues 48 to 74 and 235 to 239. A predicted amphipathic-α-helical domain is also shown. The location of these domains was estimated from the previous report by Wiethoff et al. (2005).

Twenty-one of twenty-two single-nucleotide substitutions were located in coding sequences from eight regions (E1B, E2B, L1, L2, L3, E2A, L4, E3). Ten single-nucleotide substitutions were non-synonymous substitutions, while 11 were synonymous substitutions (**Table 5**). Of the 10 non-synonymous substitutions, six were located in the pVI protein in the L3 region (**Figure 4**), which is one of the four cement proteins of HAdVs (IIIa, VI, VIII, and IX). Three of the six single-nucleotide non-synonymous substitutions (A66G, R68K, and V81F) occurred close to the N-terminal domain of protein VI, of which, two (A66G and R68K) were located within one of the two hexon binding domains. The other four non-synonymous substitutions were located in the 20kDa Protein, 55kDa Protein, Terminal Protein Precursor, and 14.9kDa Protein, respectively (**Table 5**). The 11 synonymous nucleotide substitutions were present in the 20kDa Protein, 55kDa Protein, DNA polymerase, 43kDa Protein, pV, pVI, Hexon, DNA Binding Protein, 100kDa Hexon-assembly Associated Protein, 11.7kDa Protein, and 20.1kDa Protein, respectively (**Table 5**). The capsid protein genes were highly conserved between the two strains isolated 5 years apart. There was only one single-nucleotide mutation (T to C) in the hexon gene, which resulted in a synonymous substitution (L to L). Both the penton base and fiber genes of the two strains were identical with each other.

Compared with the genome of strain QS-DLL, strain BJ01 had nucleotide insertions in three regions (TCCATATCCGTGT, AAAAAA, and A, respectively) and one deletion (AA) within L2 pX poly(A) region. All the insertions and deletions were located in non-coding regions (NCR). The insertions of “TCCATATCCGTGT” were located at the upstream of E1A poly(A) region, whereas the insertions of “AAAAAA” were located within IX poly(A) region, and the insertion of “A” were located within L2 pX poly(A) region (**Table 5**).

## Discussion

Human adenoviruses were traditionally classified primarily according to their serological profile and hemagglutination properties (Hierholzer et al., 1991), and these methods are mainly based on the interaction between antibodies and the major capsid proteins. Presently, 51 HAdV serotypes have been recognized. However, serological methods seem not appropriate to characterize adenoviruses just by the epitopes. As an example, HAdV-D15, HAdV-D29, and HAdV-D56 were found to share the same virus neutralization (VN) epitopes (Stevens et al., 1967; Wigand, 1987; Robinson et al., 2011, 2013a), but all three viruses contain divergent genomes and biological properties (Robinson et al., 2011, 2013a). Currently, more and more traditional techniques have been replaced by genomic criteria. The genome data were proposed to be used to type HAdVs (Seto et al., 2011), *i.e*., HAdVs should be identified, typed, and characterized based on the complete genome sequences instead of serological methods (Zhang et al., 2006, 2012a; Robinson et al., 2009; Walsh et al., 2010; Zhou et al., 2012; Zhang Q. et al., 2016).

HAdV-B55 was first isolated from an ARD outbreak in a military training base in 1969, then it was named HAdV-B11a by partial characterization of its hexon and fiber genes (Hierholzer et al., 1974), as well as restriction enzyme patterns (Zhang Q. et al., 2016). Until 2006, along with several HAdV-B55-associated ARD outbreaks (Hierholzer et al., 1974; Chmielewicz et al., 2005; Yang et al., 2009; Kajon et al., 2010), it was ultimately revised as HAdV-B55 by Walsh et al. (2010) after bioinformatic analysis of the complete genome of strain QS-DLL. In 2011, 5 years after the emergence of the first HAdV-B55 strain QS-DLL in China (Walsh et al., 2010), HAdV-B55 re-emerged and caused a CAP outbreak in China (Gu et al., 2012; Zhang et al., 2012a; Cao et al., 2014). Since then, there have been more than 10 HAdV-B55-associated ARD outbreaks in China, for example, in Shanxi province, Chongqing, Beijing, Jiangsu province, Hebei province, Jinan, Tianjin, and Guangdong province (Yang et al., 2009; Walsh et al., 2010; Zhang et al., 2012a; Li et al., 2014; Chen et al., 2016; Gao et al., 2016; Tan et al., 2016; Yi et al., 2017), with severe and fatal cases (Zhu et al., 2009; Cao et al., 2014; Tan et al., 2016; Zhang S.Y. et al., 2016). Similar ARD outbreaks were also reported in other countries (Geng et al., 2013; Lafolie et al., 2016; Salama et al., 2016; Park et al., 2017).

Here presented is the detailed comparative genomic analysis of the re-emergent HAdV-B55 strain BJ01 with the first isolate QS-DLL. The genomic nucleotide identity between the two strains was 99.87%, along with 26 mutations, including 11 synonymous and 10 non-synonymous substitutions, 3 insertions, one deletion and one G to A mutation in L2 NCR (**Table 5**). Compared with other HAdV types, such as HAdV-B3, -C5, -B7, -B14, -D36, of which the genomes are usually conserved and stable (Mahadevan et al., 2010; Seto et al., 2010b; Nam et al., 2014; Alkhalaf et al., 2015; Yu et al., 2016; Zhang et al., 2017), HAdV-B55 showed similarly conserved genomes when looking at the two strains isolated five years apart (2006 vs 2011). However, the major population distribution of HAdV-B55 associated disease has been changed from juveniles to adults, whereas the disease severity remains similar. The major gene mutations between the two isolates are located in the L3 pVI protein, one of the structural proteins of HAdVs, in which six single-nucleotide non-synonymous substitutions and one synonymous substitution were identified (**Figure 4**). pVI and its processed form protein VI are special proteins because they have different functions at various stages of HAdV infections. For example, during adenoviral infection, protein VI aids the virion particle to escape from the endosome by inducing a pH-independent disruption of the membrane (Wiethoff et al., 2005). The N-terminal domain in pVI with predicted amphipathic α-helical structure is required for membrane lytic activity (Wiethoff et al., 2005). Additionally, it binds the hexon protein and facilitate nuclear import of hexon (Matthews and Russell, 1995). Two regions, between amino acid residues 48–74 and 233–239 of pVI, were required for the interaction with the hexon protein, where two non-synonymous substitutions were identified (**Figure 4**). Whether the non-synonymous substitutions in pVI can speed up the adenoviral maturation and further change the population distribution of HAdV-B55 infection remains unknown. Further wet-bench confirmation is necessary.

Phylogenetic analysis of genome sequences, as well as individual genes, showed a subclade composed of HAdV-B55 isolates (BJ01 included) with high bootstrap values (**Figures 3A–D**). HAdV-B55 was closely related to HAdV-B11 (Slobitski) and HAdV-B14 (de Wit), forming a clade (**Figures 3A–D**), which was consistent with the global pairwise alignment data (**Figure 2A**). Recombination analysis further revealed the mechanism that the two loops of the HAdV-B11 hexon were embedded in the whole genome of HAdV-B14 (**Figures 2B–D**), giving evidence that the hexon gene, particularly hypervariable regions, acts as an important hotspot for genome recombination. Although the penton base and shaft/knob boundary of the fiber gene were also known to be two recombination sites (Robinson et al., 2009; Liu et al., 2011), no recombination in these regions was identified in HAdV-B55 genomes. The neutralizing and protective antibodies in human are induced primarily by the hexon protein, partially by the fiber and penton base proteins (Gahery-Segard et al., 1998; Hong et al., 2003; Sumida et al., 2005). The lack of non-synonymous substitutions in the three capsid proteins of HAdV-B55 strains make the HAdV-B55 vaccine development easier when compared with other respiratory viruses, such as influenza viruses.

Genome recombination is an important strategy driving HAdV evolution (Robinson et al., 2009, 2011; Kajon et al., 2010; Walsh et al., 2010, 2011; Liu et al., 2011, 2012; Matsushima et al., 2012), and results in the changes in pathogenicity (Walsh et al., 2009; Singh et al., 2012; Zhou et al., 2012). For HAdVs, to some extent, recombination can be defined as an adaptive change resulting from environmental pressures. In the example of HAdV-B55, the initial HAdV-B14 infection may induce neutralizing antibodies against this type. Meanwhile the co-infection and co-replication of HAdV-B11 and HAdV-B14 in the intestine may lead to the recombination between HAdV-B11 and HAdV-B14 hexon genes, *i.e.*, the resulting HAdV-B55 can escape from pre-existing immunity against HAdV-B14 from the host due to its acquisition of HAdV-B11 loops. The previous study has confirmed that the neutralizing and protective antibodies in human are induced primarily by the two hexon loops. The acquisition both loops of HAdV-B11 is beneficial to the survival and spreading of the recombinant HAdV-B55, because the previous absence of circulating HAdV-B11 in China (Han et al., 2013; Chen et al., 2016; Yu et al., 2016) led to a corresponding low level of herd immunity, which might be associated with the subsequent more and more ARD outbreaks caused by HAdV-B55 (Yang et al., 2009; Zhu et al., 2009; Walsh et al., 2010; Zhang et al., 2012a; Zhang S.Y. et al., 2016; Cao et al., 2014; Li et al., 2014; Chen et al., 2016; Gao et al., 2016; Tan et al., 2016; Yi et al., 2017). This is one of the immune escape capabilities of HAdVs. The detailed recombination mechanism of HAdV-B55 might be illustrated by *in vitro* co-infection and replication of the two types of adenoviruses, HAdV-B11 and -B14. In addition, we also infected A549, Hela, HEp-2, Hep-G2, and Vero cells with HAdV-B55, -B11, and -B14, respectively (data not shown). The replication efficiency of different types in the cells was compared. The three types of HAdVs replicated most efficiently in A549 cells. Unfortunately, no significant difference of replication efficiency among the three types of HAdVs was identified in all the tested cell clines. The prevalence and outbreak of HAdV-B55 might be not associated with the virus replication efficiency.

In summary, comparative genomic analysis of re-emergent HAdV-B55 pathogens revealed conserved capsid proteins and low non-synonymous substitutions per site of HAdV-B55, which provides the foundation for the development of effective vaccines against this pathogen. It also facilitates the further studies of the adenovirus epidemiology, molecular evolution, patterns of pathogen emergence and re-emergence (Zhang et al., 2017), and the predication of genome recombination between adenoviruses, as well as the development of public health strategies, including surveillance.

## Author Contributions

QZ, XW, and BC conceived and designed the study. ZC, YY, SJ, W-GL, W-WC, JZ, ML, ShZ, NC, JO, and SuZ performed the computational analyses and made the figures and tables. ZC, YY, and QZ wrote the manuscript. All authors reviewed the manuscript.

## Conflict of Interest Statement

The authors declare that the research was conducted in the absence of any commercial or financial relationships that could be construed as a potential conflict of interest.

## Footnotes

1 http://hadvwg.gmu.edu

2 https://sray.med.som.jhmi.edu/scroftware/simplot/

3 http://mafft.cbrc.jp/alignment/server/

4 http://genome.lbl.gov/vista/lagan/submit.shtml

5 http://www.megasoftware.net/index.html
